# The Role of the NO/cGMP Pathway and SKCa and IKCa Channels in the Vasodilatory Effect of Apigenin 7-Glucoside

**DOI:** 10.3390/molecules30214265

**Published:** 2025-10-31

**Authors:** Maria Luiza Fidelis da Silva, Erdi Can Aytar, Arquimedes Gasparotto Junior

**Affiliations:** 1 Laboratory of Cardiovascular Pharmacology (LaFaC), Faculty of Health Sciences, Federal University of Grande Dourados (UFGD), Dourados 79804-970, MS, Brazil; m.alufidelis@hotmail.com; 2 Faculty of Agriculture, Department of Horticulture, Usak University, 64200 Uşak, Türkiye; erdicanaytar@gmail.com

**Keywords:** Flavone, mesenteric vascular bed, K^+^ channels, vasodilation

## Abstract

This study aimed to elucidate the vasorelaxant mechanism of action for apigenin 7-glucoside (A7G) by integrating computational and ex vivo pharmacological approaches. Molecular docking simulations were conducted to predict the binding affinities and interactions of A7G with key vascular proteins, specifically human endothelial nitric oxide synthase (eNOS-PDB ID: 1M9M), and human intermediate (IKCa-PDB ID: 9ED1) and small-conductance (SKCa-PDB ID: 6CNN) Ca^2+^-activated K^+^ channels. The vasodilatory properties of A7G were subsequently evaluated in isolated mesenteric vascular beds (MVBs) from normotensive Wistar Kyoto (WKY) and spontaneously hypertensive rats (SHR). The in silico analysis indicated that A7G possesses favorable binding affinities for the 1M9M, 9ED1, and 6CNN protein targets. Pharmacological assessments demonstrated that A7G induced a dose- and endothelium-dependent reduction in perfusion pressure in MVBs from WKY and SHR rats. The vasodilatory response to A7G was completely abrogated by perfusion with a high-potassium solution or a non-selective K^+^ channel blocker. Furthermore, co-administration of apamin and TRAM-34, selective inhibitors of SKCa and IKCa, respectively, also abolished the vasorelaxant effects of A7G. Collectively, these findings suggest that the vascular effects of A7G in both WKY and SHR rats involve an endothelium-dependent mechanism, likely initiated by the activation of the NO/cGMP pathway, which culminates in the opening of IKCa and SKCa channels.

## 1. Introduction

Hypertension, a primary contributor to global cardiovascular mortality, is pathophysiologically characterized by endothelial dysfunction [1,2,3]. This condition impairs the regulation of vascular tone, which is normally mediated by factors such as nitric oxide (NO), prostaglandins, and endothelium-derived hyperpolarizing factor [4,5]. Consequently, compounds capable of modulating these endothelial pathways represent promising candidates for the pharmacological management of hypertension [6].

Apigenin 7-glucoside (A7G) is a plant-derived flavonoid widely distributed in the plant kingdom [7]. Its glucose moiety enhances key physicochemical properties, such as aqueous solubility and bioavailability [8]. The aglycone apigenin exhibits pleiotropic cardiovascular effects. It has been demonstrated to ameliorate endothelial dysfunction and induce vasodilation via the nitric oxide/cyclic guanosine monophosphate (NO/cGMP) signaling pathway and the activation of small- and intermediate-conductance calcium-activated potassium channels (SKCa and IKCa) [9,10]. Furthermore, apigenin may mitigate hypertension-induced vascular remodeling [11], reduce calcium influx into vascular smooth muscle cells [12], and suppress calcium-activated chloride channel currents [13].

A diverse spectrum of biological activities has been attributed to A7G, including antioxidant, anti-inflammatory [14,15], antiviral [16], hepatoprotective [17], antitumor [18,19], anxiolytic [20], neuroprotective [21], and antimicrobial effects [22]. Despite the documented vascular activities of its aglycone, the effects of A7G on peripheral vascular resistance remain poorly understood. Therefore, the present study was designed to characterize the vasodilatory effect of A7G and to elucidate its underlying molecular mechanisms in the isolated mesenteric vascular beds (MVBs) of normotensive Wistar Kyoto (WKY) and spontaneously hypertensive rats (SHR).

## 2. Results

### 2.1. Molecular Docking Analysis

The binding affinity and ligand efficiency metrics for compound A7G against the selected protein targets are summarized in Table 1. A7G exhibited a potent binding affinity for the 1M9M protein, with a binding energy of −9.7 kcal/mol, which corresponds to an estimated inhibition constant (K_i_) of 0.076 μM. The calculated efficiency scores for this complex were also notable: a ligand efficiency (LE) of 0.313, fit quality (FQ) of 0.855, and a binding efficiency index (BEI) of 0.024. In comparison, A7G demonstrated moderate and similar affinities for the 6CNN and 9ED1 targets, yielding binding energies of −8.5 kcal/mol (K_i_ = 0.581 μM) and −8.4 kcal/mol (K_i_ = 0.688 μM), respectively. The efficiency metrics for these two interactions were also comparable (LE ≈ 0.27, FQ ≈ 0.77, BEI = 0.019). Collectively, these results indicate that A7G possesses a markedly stronger binding affinity and higher ligand efficiency for the 1M9M target over 6CNN and 9ED1.

**Figure 1:**
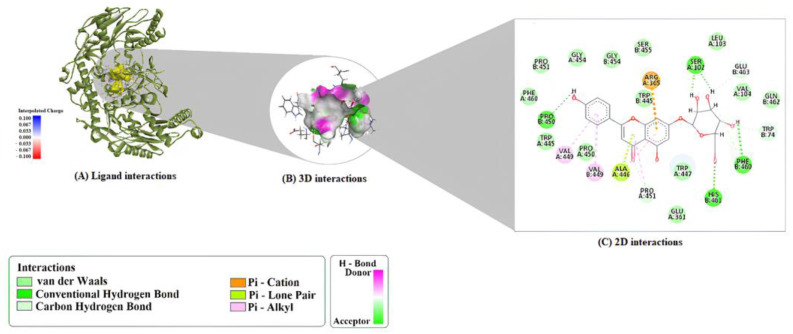
Binding mode and molecular interactions of A7G within the 1M9M active site. (**A**) 3D view of the docked complex. (**B**) 2D diagram illustrating key interactions. (**C**) 3D representation of key non-covalent contacts.

Table 2 delineates the specific molecular interactions that stabilize the binding of compound A7G to the selected protein targets. Within the active site of 1M9M, A7G established a robust network of hydrogen bonds with key residues, including ARG352, ARG189, PHE190, ARG191, LYS75, ALA184, and ASP78. The binding pose was further reinforced by π–cation (with ARG180) and π–anion (with ASP78) interactions, complemented by several hydrophobic contacts involving residues ARG189, ALA184, and LYS75 (Figure 1).

**Figure 2:**
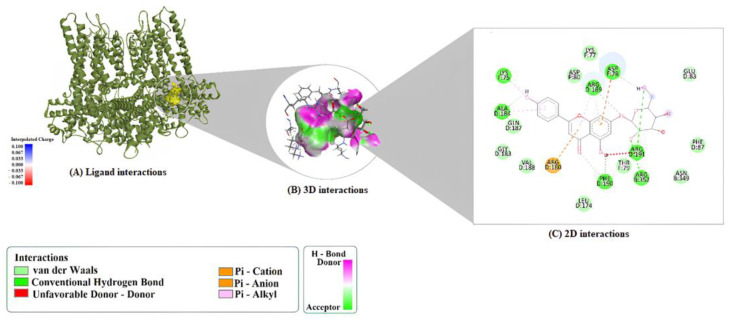
Binding interactions of A7G with the target 6CNN. (**A**) Docked complex view, (**B**) 2D interaction diagram, and (**C**) 3D interaction map showing key non-covalent contacts.

The interaction profile with 6CNN was similarly characterized by extensive hydrogen bonding with residues such as PHE190, ARG352, GLU83, and ARG189. The complex was notably stabilized by multiple π-interactions, including π–cation bonds with ARG180 and ARG189, and a π–sigma interaction involving ARG189. This residue also contributed to alkyl interactions, enhancing binding stability (Figure 2).

**Figure 3:**
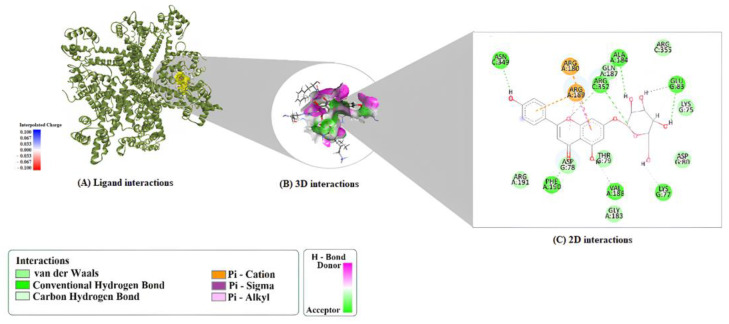
Binding interactions of A7G with the target 9ED1. (**A**) Docked complex view, (**B**) 2D interaction diagram, and (**C**) 3D interaction map showing key non-covalent contacts.

For the 9ED1 protein, A7G’s binding was mediated by hydrogen bonds with HIS461, PHE460, SER102, and TRP74, among others. This orientation was stabilized by a π–cation interaction with ARG365 and a π–lone pair interaction with ALA446. The complex was further anchored by hydrophobic contacts with ALA446, PRO451, and VAL449 (Figure 3).

Collectively, these findings reveal that A7G adopts distinct binding modes for each protein target, utilizing a diverse array of non-covalent interactions to achieve stable complex formation.

### 2.2. A7G Induces Vasodilation in the MVBs of WKY and SHR Rats

**Figure 4:**
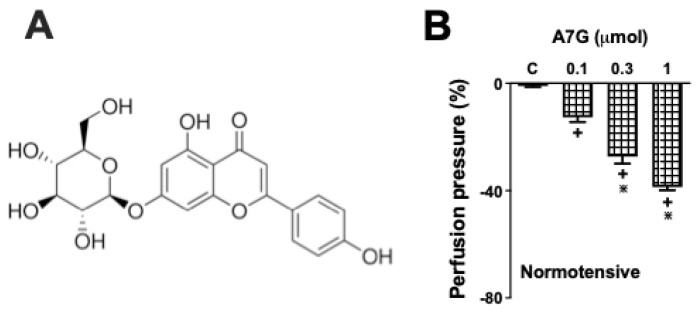
Dose-dependent vasodilation induced by apigenin 7-glucoside (A7G) in isolated mesenteric vascular beds from Wistar Kyoto (WKY) rats. Panel (**A**) shows the chemical structure of A7G. Panel (**B**) show the dose-dependent reduction in perfusion pressure in endothelium-intact preparations from WKY rats. Values are the mean ± S.E.M. of six experiments. + *p* < 0.05 vs. the previous dose; ∗ *p* < 0.05 vs. 0.03 and 0.1 µmol doses of A7G.

**Figure 5:**
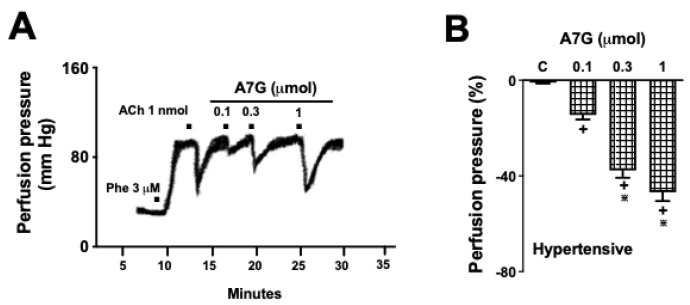
Dose-dependent vasodilation induced by apigenin 7-glucoside (A7G) in isolated mesenteric vascular beds from spontaneously hypertensive rats (SHR). Panel (**A**) shows a representative original recording of the MVB perfusion pressure in SHR, demonstrating the effects of acetylcholine (ACh) and A7G. Panel (**B**) show the dose-dependent reduction in perfusion pressure in endothelium-intact preparations from SHR. Values are the mean ± S.E.M. of six experiments. + *p* < 0.05 vs. the previous dose; ∗ *p* < 0.05 vs. 0.03 and 0.1 µmol doses of A7G.

The mean systolic blood pressure, diastolic blood pressure, and heart rate obtained from the WKY rats before the removal of the MVBs were 119 ± 8.6 mm Hg, 79 ± 4.2 mm Hg, and 279 ± 22.7 bpm, respectively (*n* = 6 per experimental group); while for the SHR, the estimated values were 159 ± 4.6 mm Hg, 102 ± 7.7 mm Hg, and 355 ± 34.4 bpm, respectively (*n* = 6 per experimental group). The MVB preparations from WKY and SHR rats exhibited a mean basal perfusion pressure of 33 ± 5.2 mm Hg and 39 ± 6.1 mm Hg, respectively, when perfused with physiological salt solution (PSS) at 4 mL/min. Subsequently, a stable tonic contraction was induced with 3 µM phenylephrine (Phe), increasing the perfusion pressure to a plateau of 108 ± 12.3 mm Hg for WKY and 116 ± 11.6 mm Hg for SHR rats. Under these conditions, subsequent administration of A7G (0.1, 0.3, and 1 µmol) (Figure 4A) in MVBs from WKY rats resulted in a significant dose-dependent vasodilation, with percentage reductions in perfusion pressure of 12 ± 2.5, 27 ± 4.2, and 38 ± 5.1%, respectively (Figure 4B), while in SHR animals (Figure 5A,B) the percentage reduction in perfusion pressure was 15 ± 3.1, 37 ± 5.5, and 47 ± 6.7%, respectively. The maximal effect, observed with 1 µmol of A7G, were similar to the response elicited by 1 nmol of acetylcholine (ACh), a reference vasodilator, reducing the perfusion pressure by approximately 40%.

### 2.3. Involvement of the Vascular Endothelium and the Nitric Oxide (NO)-Soluble Guanylate Cyclase (sGC)-Cyclic Guanosine Monophosphate (cGMP) Pathway in the A7G-Induced Vasodilation in the MVBs of WKY and SHR Rats

**Figure 6:**
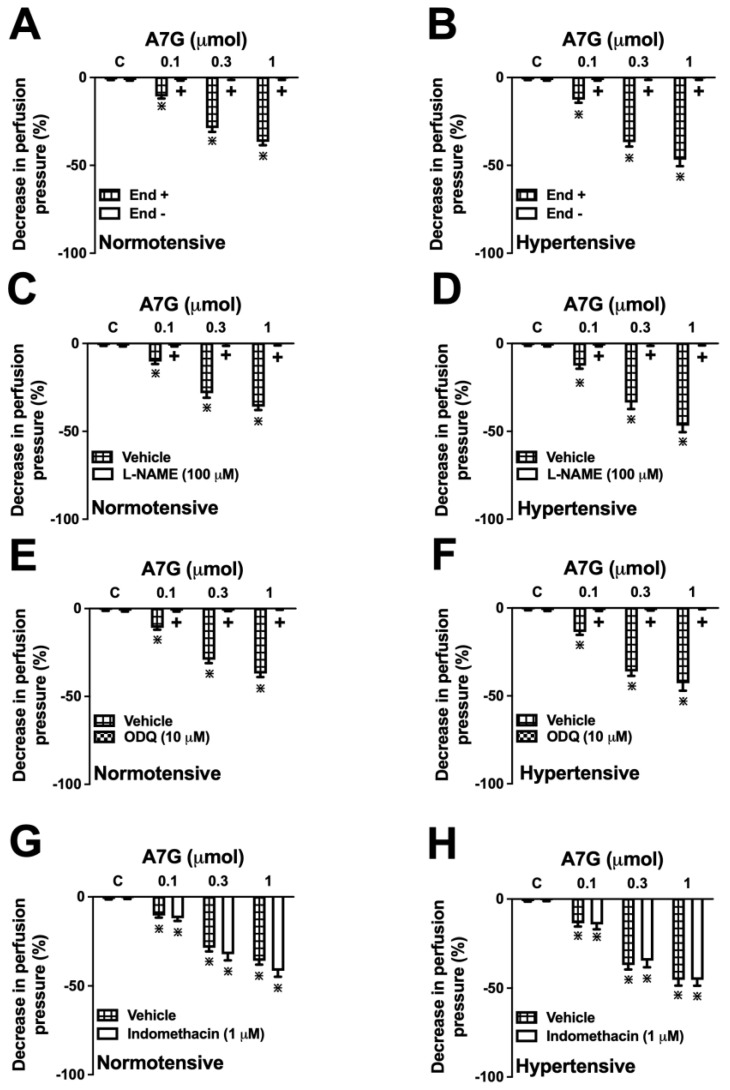
Role of the vascular endothelium and the nitric oxide -soluble guanylate cyclase-cyclic guanosine monophosphate pathway in the vasodilator effect induced by apigenin 7-glucoside (A7G) in the mesenteric vascular beds of Wistar Kyoto (normotensive) and spontaneously hypertensive rats (hypertensive). The data show the effects of A7G (0.1, 0.3, and 1 µmol) in preparations with intact (End +) versus denuded (End −) endothelium (**A**,**B**). The effects were also evaluated in endothelium-intact preparations pre-incubated for 15 min with Nω-Nitro-L-arginine methyl ester (L-NAME) (100 µM; (**C**,**D**)), [1,2,4]oxadiazolo[4,2-alpha]quinoxalin-1-one (ODQ) (10 µM; (**E**,**F**)), or indomethacin (1 µM; (**G**,**H**)). Data are expressed as the mean ± S.E.M. (*n* = 6). + *p* < 0.05 compared to the respective control group (End + in panel (**A**); Vehicle in panels (**B**–**D**)). * *p* < 0.05 compared to the preceding dose.

The vasodilator responses to A7G (0.1, 0.3, and 1 µmol) in MVBs from normotensive and hypertensive rats were demonstrated to be endothelium-dependent. Chemical denudation of the endothelium with sodium deoxycholate virtually abolished the A7G-induced vasodilation by 99% in WKY (Figure 6A) and SHR (Figure 6B) rats, an efficacy similar to that observed with acetylcholine (ACh).

In both normotensive and hypertensive rats, the vasodilator effect of A7G was completely abrogated in the presence of either the nitric oxide synthase (NOS) inhibitor, L-NAME (Figure 6C,D), or the soluble guanylate cyclase (sGC) inhibitor, ODQ (Figure 6E,F). Conversely, pre-treatment with the cyclooxygenase (COX) inhibitor, indomethacin, did not significantly alter the A7G-induced vasodilation in either WKY or SHR rats (Figure 6G,H), thereby ruling out the involvement of prostaglandins in this response.

Taken together, these findings indicate that the vasodilator effect of A7G is mediated entirely by the endothelium-derived NO/sGC/cGMP pathway in both models used.

### 2.4. Involvement of K^+^ Channels in the A7G-Induced Vasodilation in the MVBs of WKY and SHR Rats

**Figure 7:**
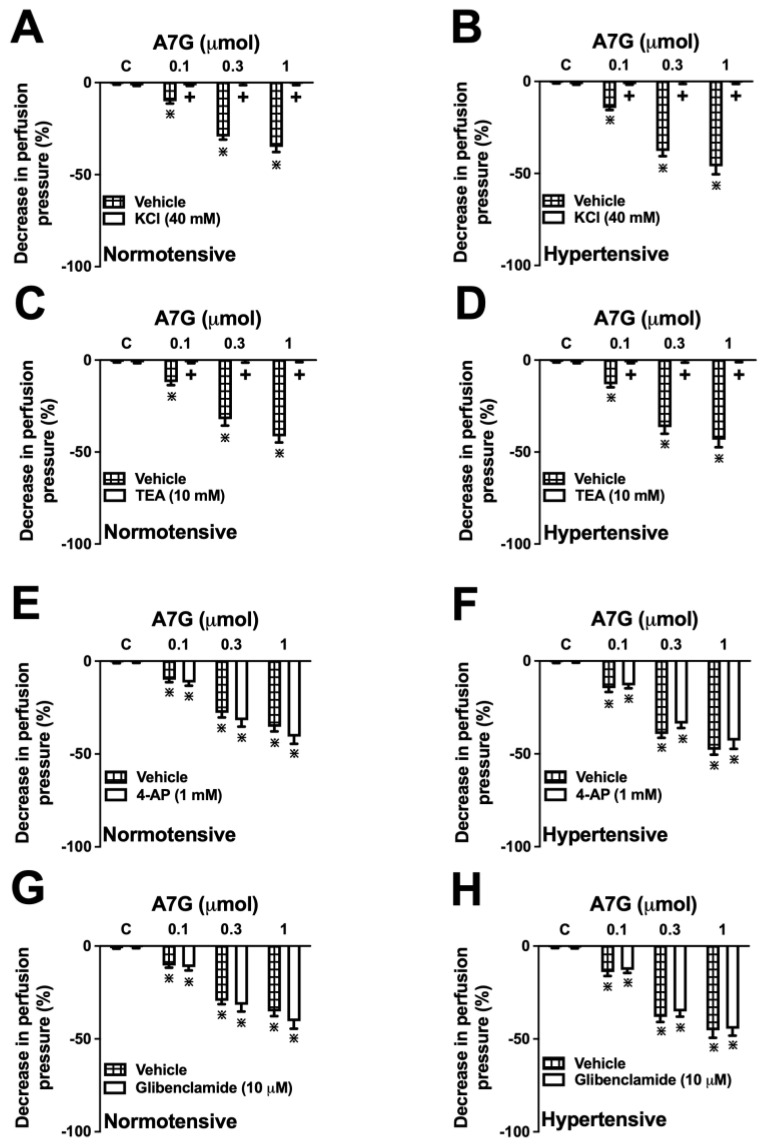
Involvement of K^+^ channels in the A7G-induced vasorelaxant response in the mesenteric vascular beds of Wistar Kyoto (normotensive) and spontaneously hypertensive rats (hypertensive). The panels illustrate the vasorelaxant effect of A7G in a high K^+^ medium (40 mM KCl; (**A**,**B**)), or in the presence of the non-specific K^+^ channel inhibitor tetraethylammonium (TEA, 10 mM; (**C**,**D**)), the KV channel inhibitor 4-aminopyridine (4-AP, 100 µM; (**E**,**F**)), or the KATP channel inhibitor glibenclamide (10 µM; (**G**,**H**)). All data are expressed as the mean ± S.E.M. from 6 preparations per group. + *p* < 0.05 denotes a significant difference from the vehicle group; * *p* < 0.05 denotes a significant difference from the previous dose.

The vasodilator responses to A7G (0.1, 0.3, and 1 µmol) in MVBs from Wistar Kyoto and SHR rats were demonstrated to be potassium channel-dependent. The vasodilator response induced by all doses of A7G was abolished in a depolarizing medium containing 40 mM KCl (Figure 7A,B) and in the presence of the non-specific K^+^ channel blocker tetraethylammonium (Figure 7C,D). Conversely, the pharmacological inhibition of ATP-sensitive K^+^ channels (KATP) with glibenclamide (Figure 7E,F) or voltage-gated K^+^ channels (Kv) with 4-aminopyridine (Figure 7G,H) did not modify the vasodilatory effect of A7G.

### 2.5. The Vascular Effect of A7G Is Mediated by the Activation of Intermediate-(IKCa) and Small-Conductance (SKCa) Ca^2+^-Activated K^+^ Channels in MVBs from WKY and SHR Rats

**Figure 8:**
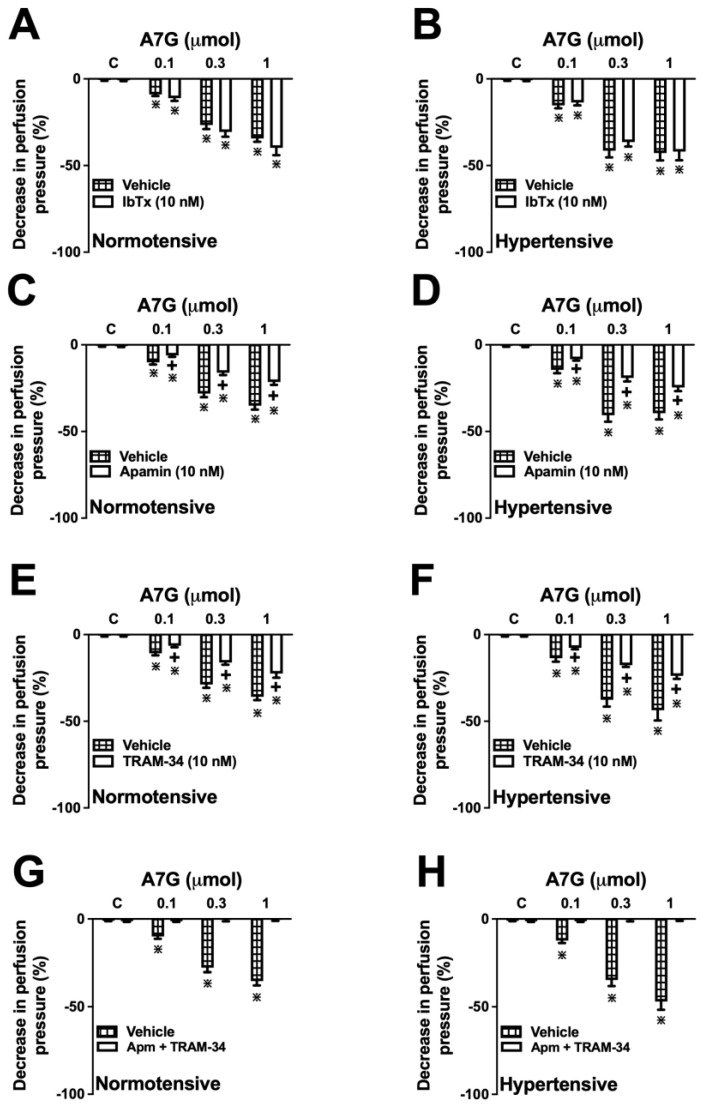
Role of intermediate- (IKCa) and small-conductance (SKCa) Ca^2+^-activated K^+^ channels in the vasodilatory effect of A7G in Wistar Kyoto (normotensive) and spontaneously hypertensive rats (hypertensive). The panels show the vasorelaxant effect of A7G in the absence (vehicle) or presence of Iberiotoxin (IbTx, 10 nM), a selective large-conductance Ca^2+^-activated K^+^ (BKCa) channel blocker (**A**,**B**), apamin (10 nM), a selective small-conductance Ca^2+^-activated K^+^ (SKCa) channel blocker (**C**,**D**), TRAM-34 (10 nM), a selective intermediate-conductance Ca^2+^activated K^+^ (IKCa) channel blocker (**E**,**F**); or a combination of apamin and TRAM-34 (**G**,**H**). Values are expressed as the mean ± S.E.M. (*n* = 6). + *p* < 0.05 vs. vehicle group; * *p* < 0.05 vs. previous dose.

Prior perfusion with iberiotoxin (IbTx, 10 nM), a selective large-conductance Ca^2+^-activated K^+^ (BKCa) channel blocker, did not affect the vasodilatory effects induced by A7G in WKY and SHR animals (Figure 8A,B). Conversely, pre-incubation with either TRAM-34, a selective IKCa channel blocker, or apamin, a selective SKCa channel blocker, significantly attenuated the vasodilation induced by all A7G doses in both normotensive and hypertensive rats (Figure 8C–F). Furthermore, the co-administration of TRAM-34 and apamin completely abolished the vasorelaxant response induced by A7G in both animal models (Figure 8G,H).

## 3. Discussion

It is well-established that flavanols, a class of flavonoids analogous to A7G, can induce arteriolar vasodilation by triggering the release of endothelial mediators such as NO and prostacyclin (PGI_2_), and through endothelium-dependent hyperpolarization [23,24,25]. Furthermore, several studies indicate that the aglycone apigenin can reduce endothelial dysfunction due to its antioxidant action [11] and induce vasodilation via mechanisms that include the release of endothelial NO, a reduction of calcium influx into vascular smooth muscle, and the activation of KCa channels [10,13].

Building on the documented cardiovascular effects of apigenin, we postulated that its glycoside, A7G, would similarly influence vascular tone. Accordingly, the initial phase of our investigation utilized molecular docking to predict the binding affinities and interaction patterns between A7G and key vascular target proteins. Our in silico analysis indicates that A7G establishes high-affinity, selective complexes with both endothelial nitric oxide synthase (eNOS, 1M9M) and the Ca^2+^-activated K^+^ channels IKCa (9ED1) and SKCa (6CNN). The stability of these complexes is attributed to a synergistic network of non-covalent interactions. Collectively, these computational data characterize A7G as a potent triple action modulator, with the predicted capacity to concurrently engage eNOS and promote the opening of IKCa and SKCa channels.

To validate the in silico findings within a complex biological model, we utilized mesenteric vascular beds (MVBs) isolated from spontaneously hypertensive rats (SHR). The SHR model is ideal for evaluating the pharmacological effects of A7G, as these animals exhibit alterations in endothelial function and ion channel expression associated with hypertension [26,27]. Our results demonstrate that A7G induces a significant and dose-dependent vasodilator effect in resistance arteries from both SHR and normotensive controls. We also confirmed that the integrity of the vascular endothelium is indispensable for the vasodilatory effects induced by A7G. Furthermore, we evidenced that the vasodilator response to A7G is directly dependent on the nitric oxide (NO) signaling cascade. Pharmacological blockade with either the NO synthase inhibitor L-NAME or the soluble guanylate cyclase inhibitor ODQ completely abolished the A7G-induced vasodilation. These findings unequivocally implicate the endothelium and the subsequent activation of the NO/cGMP pathway as the primary mediator of A7G’s effect.

Based on the preceding in silico results, we proceeded to investigate the role of potassium channels in the vasodilatory action of A7G. The rationale for this was the established role of K^+^ channel-induced hyperpolarization as a key downstream effector mechanism of the NO/cGMP pathway in vascular smooth muscle [28,29]. Our experiments confirmed that the A7G-induced vasodilation in MVBs is indeed dependent on the activation of vascular smooth muscle K^+^ channels. This conclusion is supported by the complete elimination of the vasodilator response under conditions of high extracellular K^+^ (40 mM KCl) and in the presence of the non-selective K^+^ channel blocker, tetraethylammonium [30,31,32]. To delineate the specific channel subtypes responsible for this effect, we conducted further pharmacological blockade experiments. We found that the inhibition of either ATP-sensitive K^+^ (KATP) channels with glibenclamide or voltage-gated K^+^ (Kv) channels with 4-aminopyridine did not alter the vascular response to A7G, thereby ruling out their involvement. However, the combined blockade of intermediate-conductance (IKCa)) and small-conductance (SKCa) calcium-activated potassium channels with TRAM-34 and apamin, respectively, completely nullified the vasodilatory effect. This finding pinpoints the dual activation of IKCa and SKCa channels as the crucial terminal event in the mechanism of A7G-mediated vasodilation in SHR. This proposed framework is consistent with established evidence that flavonoids, including the aglycone apigenin, can induce the calcium-dependent activation of endothelial NO synthase (eNOS). This rise in [Ca^2+]^i can simultaneously activate eNOS, leading to the NO/cGMP signalling pathway while also directly stimulating the opening of KCa channels, including SKCa and IKCa [33].

Despite the novelty of our findings regarding the vasodilator potential of A7G, a key limitation of this study must be acknowledged. It remains uncertain whether the observed pharmacological effects are attributable directly to A7G or exclusively to its aglycone following hydrolysis. This possibility could explain the high degree of similarity between the present data and our group’s previous findings on apigenin [10]. Further investigation, specifically through preclinical pharmacokinetic and metabolomic studies, is necessary to differentiate between these two mechanisms. Such research would clarify if the observed effects on mesenteric vascular beds are caused by A7G itself or are solely due to its aglycone, apigenin.

In summary, the present study establishes A7G as a potent vasodilator within the resistance vasculature of hypertensive rats. Furthermore, we elucidate for the first time its mechanism of action, which is dependent on the vascular endothelium. Our findings indicate that A7G-induced vasodilation is mediated by the NO/cGMP pathway, which subsequently triggers the opening of SKCa and IKCa channels in arteriolar smooth muscle cells.

## 4. Materials and Methods

### 4.1. Molecular Docking Studies

To investigate the binding interactions of A7G, molecular docking simulations were performed. The three-dimensional (3D) structure of the ligand was retrieved from the PubChem database and subsequently subjected to energy minimization to obtain an energetically favorable conformation. The atomic coordinates for the target proteins were obtained from the RCSB Protein Data Bank, corresponding to the crystal and cryo-EM structures of human endothelial nitric oxide synthase (eNOS; PDB ID: 1M9M), the human SK4/calmodulin channel (KCa2.3; PDB ID: 6CNN), and the human KCa3.1/calmodulin channel (PDB ID: 9ED1).

Protein structures were prepared for docking using AutoDock Tools v1.5.7. This procedure involved the removal of crystallographic water molecules and non-standard residues, the addition of polar hydrogens, and the assignment of Gasteiger partial charges to each atom.

Docking simulations were executed using AutoDock Vina, selected for its balance of computational efficiency and scoring accuracy. A grid box was defined to fully encompass the active/binding site of each protein, allowing for a comprehensive conformational sampling of the ligand. For each target, the docked conformation exhibiting the lowest (most favorable) binding energy was selected for further evaluation [34].

### 4.2. Pharmacological Assays

#### 4.2.1. Drugs and Reagents

The following drugs, salts, and solutions were used in this study. Xylazine and ketamine hydrochloride were obtained from Syntec (São Paulo, SP, Brazil). Heparin was purchased from Hipolabor Pharmaceutical (Belo Horizonte, MG, Brazil). All other reagents were acquired from Sigma-Aldrich (Saint Louis, MO, USA), including: acetylcholine chloride, phenylephrine, indomethacin, glibenclamide, Nω-Nitro-L-arginine methyl ester (L-NAME), 1H-[1,2,4]oxadiazolo[4,2-alpha]quinoxalin-1-one (ODQ), tetraethylammonium bromide, iberiotoxin, TRAM-34, apamin, sodium deoxycholate, A7G, NaCl, KCl, NaHCO_3_, MgSO_4_, CaCl_2_, KH_2_PO_4_, dextrose, and ethylenediaminetetraacetic acid (EDTA).

#### 4.2.2. Animals

All experimental procedures were approved by the Institutional Ethics Committee of the Federal University of Grande Dourados (UFGD; approval date: 24 June 2025; protocol: CONCEA/MCTI/0301002024/PESQUISA/000003/2025) and conducted in accordance with the Guidelines for the Care and Use of Laboratory Animals, as published by the U.S. National Institutes of Health. Fourteen-week-old male Wistar and spontaneously hypertensive rats (SHR) (300–320 g) were obtained from the central animal facility at UFGD. The animals were housed under controlled environmental conditions (temperature of 22 ± 2 °C; humidity of 50 ± 10%) and maintained on a 12 h light/dark cycle (lights on at 07:00 AM). Standard rodent chow and water were provided ad libitum. Noninvasive SBP was measured using a digital tail-cuff plethysmograph equipped with a rat heater (Bonther Equipamentos & Tecnologia, Ribeirão Preto, SP, Brazil). The animals were acclimated to the procedure, and their SBP was monitored for two weeks prior to the start of the experiments.

#### 4.2.3. Mesenteric Vascular Bed Preparation

Following intraperitoneal anesthesia with ketamine (100 mg/kg) and xylazine (20 mg/kg), the MVB was isolated as previously described [35]. The tissue was immediately mounted in a water-jacketed organ bath and perfused at a constant flow rate of 4 mL/min with PSS. The PSS was maintained at 37 °C and continuously aerated with a carbogenic mixture (95% O_2_/5% CO_2_). After an equilibration period of 60 min, the viability of the preparation was confirmed by observing a vasoconstrictor response to a bolus injection of KCl (120 mM). Changes in perfusion pressure (mm Hg) were continuously recorded using a pressure transducer connected to a PowerLab^®^ data acquisition system and analyzed with Chart v7.1 software (AD Instruments, Castle Hill, Australia).

#### 4.2.4. Investigation of the Mechanisms Underlying A7G-Induced Vasodilation

To establish a dose–response relationship in the absence and presence of hypertension, MVBs with functional endothelium, obtained from both SHR and Wistar rats, were pre-constricted by continuous perfusion with PSS containing 3 µM Phe. Once a stable perfusion pressure was reached, doses of A7G (0.03, 0.1, 0.3, and 1 µmol) were injected into the system at 3 min intervals, and the resulting changes in perfusion pressure were recorded.

Since the vasodilator response to A7G was similar in both normotensive and hypertensive animals, the underlying pharmacological mechanisms were investigated in WKY and SHR rats. This model was selected given the therapeutic relevance of vasodilator drugs for the treatment of hypertension. To determine the contribution of the endothelium, a separate set of MVBs underwent endothelial denudation via a 30 s perfusion with sodium deoxycholate (1.8 mg/mL). After a 40 min stabilization period, the successful removal of functional endothelium was confirmed by the lack of a vasodilatory response to acetylcholine (ACh, 1 nmol). Subsequently, a dose–response curve to A7G (0.1, 0.3, and 1 µmol) was constructed in these endothelium-denuded preparations.

To investigate the signaling pathways underlying the vasodilation induced by A7G, endothelium-intact mesenteric vascular beds were pre-constricted with Phe (3 µM) and continuously perfused with one of the following pharmacological inhibitors, as previously described by da Silva et al. [36].

To investigate the NO/cGMP pathway,

1. L-NAME (100 µM), a non-selective NOS inhibitor;
2. Indomethacin (1 µM), a non-selective COX inhibitor;
3. ODQ (10 µM), a selective inhibitor of sGC.

To investigate the role of K^+^ channels and membrane depolarization:

1. KCl (40 mM);
2. TEA (10 mM), a non-specific K^+^ channel blocker;
3. Glibenclamide (10 µM), a KATP channel blocker;
4. 4-Aminopyridine (100 µM), a Kv channel blocker;
5. IbTX (10 nM), a BKCa channel blocker;
6. TRAM-34 (10 nM), a IKCa channel blocker;
7. Apamin (10 nM), a SKCa channel blocker.

Subsequently, the dose–response curve to A7G (0.1, 0.3, and 1 µmol) was repeated.

### 4.3. Statistical Analysis

All data are expressed as the mean ± standard error of the mean (SEM) for six independent experiments per group (*n* = 6). Statistical analyses were performed using GraphPad Prism 10.6.0 for macOS (GraphPad Software, Boston, MA, USA). Comparisons between groups were made using a one-way analysis of variance (ANOVA) followed by Bonferroni’s post hoc test, or by an unpaired Student’s *t*-test, where appropriate. Differences were considered statistically significant at *p* < 0.05.

## 5. Conclusions

Our findings suggest that the vascular effects of A7G in both WKY and SHR rats involve an endothelium-dependent mechanism, likely initiated by the activation of the NO/cGMP pathway, which culminates in the opening of IKCa and SKCa channels.

## Figures and Tables

**Table 1:** Calculated binding affinities and efficiency metrics for A7G with selected target proteins.

Compound	Protein	Binding Energy (kcal/mol)	LE	FQ	BEI	Estimated K_i_ (μM)
A7G	1M9M	−9.7	0.313	0.855	0.024	0.076
A7G	6CNN	−8.5	0.274	0.776	0.019	0.581
A7G	9ED1	−8.4	0.271	0.767	0.019	0.688

BEI: Binding Efficiency Index; FQ: Fit Quality; K_i_: Estimated Inhibition Constant; LE: Ligand Efficiency.

**Table 2:** Molecular interactions of A7G with target proteins.

Compound	Protein	H-Bond	Pi–Pi Stacking	Alkyl Interactions
A7G	1M9M	ARG352:HH12–O4 ARG189:HH21–O5 PHE190:HN–O7 ARG191:HH12–O10 ARG191:HH22–O10 LYS75:HZ3–O9 H4–PHE190:O H7–ALA184:O H17–ASP78:OD2	ARG180 (π–Cation) ASP78 (π–Anion)	ARG189 (π–Alkyl × 2) ALA184 (π–Alkyl) LYS75 (π–Alkyl)
A7G	6CNN	PHE190:HN–O7 ARG352:HH21–O1 ARG352:HH22–O6 H1–GLU83:OE1 H2–GLU83:OE1 H3–ALA184:O H4–VAL188:O H7–ASN349:OD1 H17–LYS77:O H16–GLU83:OE1 (C-H bond) ARG189:NH2	ARG180 (π–Cation) ARG189 (π–Cation; π-Donor H-Bond) ARG189:HG1 (π–Sigma)	ARG189 (π–Alkyl)
A7G	9ED1	HIS461:HD1–O10 H1–PHE460:O H2–SER102:O H3–SER102:O H4–O7 H7–PRO450:O PRO451:HD2–O7 (C–H) TRP74:HD1–O2 (C–H) GLU463:HA–O3 (C–H) H14–SER102:O (C–H) H15–PHE460:O (C–H) H20–PHE460:O (C–H)	ARG365 (π–Cation) ALA446 (π–Lone Pair)	ALA446 (π–Alkyl) PRO451 (π–Alkyl) VAL449 (π–Alkyl) VAL449 (π–Alkyl, chain B)

## Data Availability

The raw data supporting the conclusions of this article will be made available by the authors on request.

## Abbreviations

CVDs	Cardiovascular diseases
A7G	Apigenin 7-glucoside
eNOS	Endothelial Nitric Oxide Synthase
BKCa	Large-conductance Calcium-activated Potassium Channel
Kv	Voltage-gated K^+^ channels
KATP	ATP-sensitive potassium channels
MVBs	Mesenteric Vascular Beds
cGMP	Cyclic Guanosine Monophosphate
ACh	Acetylcholine
L-NAME	Nω-Nitro-L-arginine methyl ester
PGI_2_	Prostacyclin
TEA	Tetraethylammonium
IbTX	Iberiotoxin
PSS	Physiological Saline Solution
SHR	Spontaneously Hypertensive Rats
FQ	Fit Quality
LE	Ligand Efficiency
BEI	Binding Efficiency Index
Ki	Inhibition Constant
ANOVA	Analysis of Variance
ATP	Adenosine Triphosphate
SKCa	Small-conductance Calcium-activated Potassium Channel
IKCa	Intermediate-conductance Calcium-activated Potassium Channel
PDB	Protein Data Bank
TRAM-34	Selective IKCa channel blocker
ODQ	1H-[1,2,4]oxadiazolo[4,2-alpha]quinoxalin-1-one
